# Functional Significance of Labellum Pattern Variation in a Sexually Deceptive Orchid (*Ophrys heldreichii*): Evidence of Individual Signature Learning Effects

**DOI:** 10.1371/journal.pone.0142971

**Published:** 2015-11-16

**Authors:** Kerstin Stejskal, Martin Streinzer, Adrian Dyer, Hannes F. Paulus, Johannes Spaethe

**Affiliations:** 1 Department of Integrative Zoology, Faculty of Life Sciences, University of Vienna, Vienna, Austria; 2 Department of Behavioral Physiology and Sociobiology, Biozentrum, University of Wuerzburg, Würzburg, Germany; 3 current address: Department of Neurobiology, Faculty of Life Sciences, University of Vienna, Vienna, Austria; 4 Department of Physiology, Monash University, Clayton, Australia; 5 School of Media and Communication, RMIT University, Melbourne, Australia; Indian Institute of Science, INDIA

## Abstract

Mimicking female insects to attract male pollinators is an important strategy in sexually deceptive orchids of the genus *Ophrys*, and some species possess flowers with conspicuous labellum patterns. The function of the variation of the patterns remains unresolved, with suggestions that these enhance pollinator communication. We investigated the possible function of the labellum pattern in *Ophrys heldreichii*, an orchid species in which the conspicuous and complex labellum pattern contrasts with a dark background. The orchid is pollinated exclusively by males of the solitary bee, *Eucera berlandi*. Comparisons of labellum patterns revealed that patterns within inflorescences are more similar than those of other conspecific plants. Field observations showed that the males approach at a great speed and directly land on flowers, but after an unsuccessful copulation attempt, bees hover close and visually scan the labellum pattern for up to a minute. Learning experiments conducted with honeybees as an accessible model of bee vision demonstrated that labellum patterns of different plants can be reliably learnt; in contrast, patterns of flowers from the same inflorescence could not be discriminated. These results support the hypothesis that variable labellum patterns in *O*. *heldreichii* are involved in flower-pollinator communication which would likely help these plants to avoid geitonogamy.

## Introduction

Pollination by sexual deception is a rare and remarkable strategy in angiosperms as it constitutes an example of extreme floral specialization [1]. The majority of sexually deceptive plants are orchid species, although some cases have been reported for other plant families [2–4]. Whilst on a global basis, sexual deception is a rare strategy for plant pollination, it is interesting that almost all species of the Mediterranean orchid genus *Ophrys*, which comprises more than 250 species, achieve pollination by exploiting the mate-seeking behavior of insect males [5–8]. The flowers imitate olfactory, visual and tactile signals of receptive females to attract males and provoke them to land on the labellum. During the so called pseudocopulation, the deceived males try to copulate with the labellum and in this process the pollinia are attached onto the males’ body. During a subsequent visit by a male insect to another conspecific flower, the pollinia can then come into contact with the stigma, facilitating pollination [4,7,9–15]. As the mating attempts are unsatisfactory for the bee, the male’s interest to visit other flowers usually decreases after a few minutes [6,13,14,16–18].

In the mating behavior of hymenopterans, which are the most frequent pollinators in the genus *Ophrys* [6], olfaction is the key sensory modality involved in male-female communication [19]. In order to attract only conspecific males, the sex pheromones produced by females are highly specific [4,20,21]. Due to the high specificity ensured by the imitated sex pheromones, visual signals were assumed to only play a minor role in pollinator attraction, and thus most *Ophrys* species are found to be visually inconspicuous and mainly possess flowers with achromatic cues to attract potential pollinators [8,15,22]. Recently, however, it was shown that some *Ophrys* species enhance pollinator attraction by means of color signals [23–25], and in addition to chromatic cues, some *Ophrys* species possess bright and highly complex line patterns that contrast well to the dark labellum [22,26]. Currently, the role of such patterns in some *Ophrys* species is poorly understood.

Flower patterns are widespread in angiosperms and show high variation in form and function. Some markings raise the effectiveness of flower visitation by guiding the visitors to the often elusive nectaries [27–30] or increase the tendency of regular flower visits instead of nectar robbing [31]. Other markings imitate pollen, anthers or stamen to attract pollinators without exposing the real pollen to environmental conditions [32,33] or simulate higher amounts of pollen to increase attractiveness to pollinators [34]. Additionally, some patterns serve as enticement for mate-seeking male insects [3,35]. However, many flower patterns are small and delicate, and their functional significance remains unknown. Moreover, the spatial resolving power of insect pollinators’ compound eyes is typically considered to be relatively low [36], especially compared to the resolving power of a lens eye for viewing stimuli at a distance [37]. Thus it is not clear in many cases how and at what distance flower patterns are perceived by the pollinators [38].

In *Ophrys*, labella of the *O*. *oestrifera* and *O*. *heldreichii* group possess bright and highly complex patterns on a dark background [22]. It was supposed that these patterns mimic the wings or body-markings of the pollinator’s females [6] but recently, Streinzer et al. [26] showed that during their approach to *O*. *heldreichii* flowers, the pollinators, males of the long-horned bee *Eucera berlandi*, do not discriminate between flowers with or without a pattern on their labellum as long as the olfactory signal is present [26]. An alternative hypothesis suggests that the labellum pattern might be learned by the unsatisfied males to avoid re-visitations [14]. As a consequence, to ensure subsequent visits by the males and thus successful pollen transfer, individual plants within a population are selected to possess distinct patterns to evade the association built between the negative experience of a male after an unsuccessful copulation and the corresponding pattern. This idea is supported by the observation that the labellum pattern of flowers from different plants appears distinct, whereas patterns from flowers of a single inflorescence have been reported to be more similar [14]. Recent work also shows that the sensory processing capabilities of bees have the capacity to use negative associations to promote high levels of perceptual learning within individual subjects, depending upon experience with visual stimuli and potential allocation of selective attention [39]. However, currently we are not aware of any systematic investigation and quantification of pattern similarity/dissimilarity within and between inflorescences in the *O*. *oestrifera* and *O*. *heldreichii* group, nor if the visual processing of insects like bees is in principle able to discriminate between natural complex labellum patterns.

Our study thus aims to quantify the variation of labellum patterns within and between populations of *O*. *heldreichii* and test if bees can learn the patterns during their flower visit. In particular, we address the following questions:

1. How similar/different are labellum patterns among flowers of the same plant, among different plants within a population, and among different populations? We expect a higher similarity of patterns among flowers of the same plant than among different plants. We measured degree of pattern overlap, degree of symmetry, pattern surface area and contour density (defined as the ratio between contour length and the area of the pattern).
2. Do males attracted by a flower pay attention to the labellum pattern? We anticipate that the males attempt to learn and memorise the pattern after negative, non-rewarding encounters. We measured the time a male hovers in front of a flower before and after a copulation event, and calculated the mean distance to the labellum to estimate spatial resolution.
3. Are bees able to discriminate and learn patterns from flowers of either the same or different conspecific plants? We hypothesize that bees are able to discriminate patterns from flowers of different plants but fail to learn patterns of flowers of the same plant.

To answer these questions we observed and filmed behavioural sequences during pseudocopulation of *Eucera berlandi* male bees interacting with *Ophrys* flowers. In addition, we tested honeybees as an accessible classic model of insect and pollinator perceptual learning using appetitive-aversive differential conditioning experiments.

## Materials and Methods

### Study species

The genus *Ophrys* comprises c. 250 species and has its center of distribution in the Mediterranean region. Heldreich’s bee orchid, *Ophrys heldreichii* SCHLECHTER, is widespread and abundant on Crete and possesses a labellum pattern which is characteristic for the *O*. *heldreichii* group [22], and was thus used in this study. Its pollinators are males of the long-horned bee *Eucera (Synhalonia) berlandi* DUSMET (Apoidea, Apidae, Eucerini) [6,25].

No specific permit was required since the locations of our field study were publicly accessible and only non-invasive techniques (filming and photographing) were applied.

### Pattern analysis for quantification of similarity

**Fig 1:**
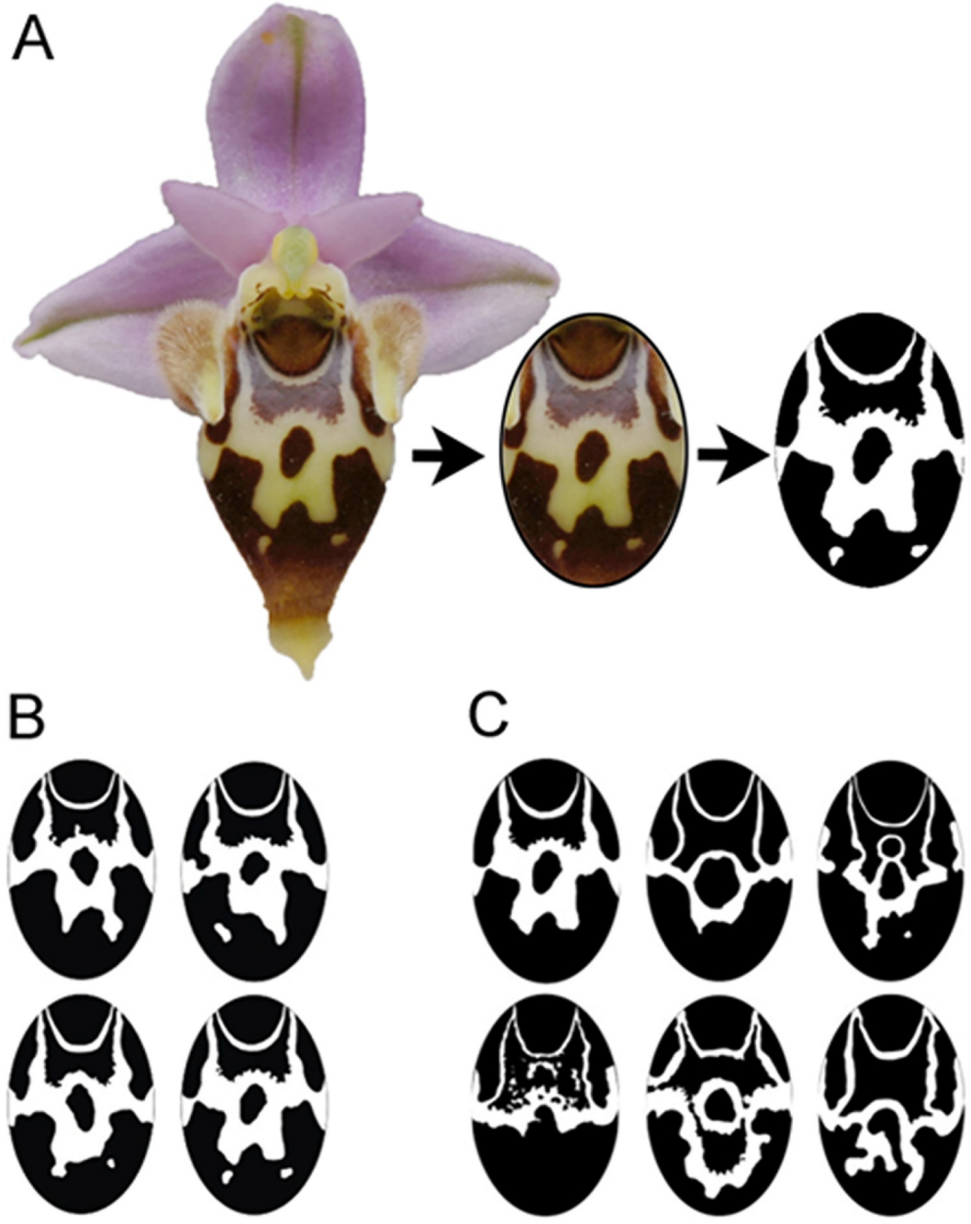
Labellum patterns of *O*. *heldreichii* flowers. (A) Transformation of a flower pattern to a black and white replica in an elliptical form used for discrimination experiments with honeybees. Patterns from flowers of the same inflorescence (B) appear more similar to each other than patters from flowers of different plant individuals (C).

To check if patterns of flowers from the same inflorescence are more similar than those of various plants, three populations of *O*. *heldreichii*, 15–17 plants per population, were investigated. Each of the inflorescences possessed 3–4 flowers, resulting in a total of 161 patterns suitable for analysis. Photographs of the labellum patterns were taken with a digital camera (Nikon D70s, Chiyoda, Japan) and modified in Adobe Photoshop CS4 by extending respective images to equal size (Fig 1A). The shape of each pattern was traced, and converted to an oval black and white picture using CorelDraw(R) Graphic Suite X3 and Image J 1.44 (Fig 1A). To quantify pattern similarity, patterns were randomly arranged in pairs and the following features were measured and compared: degree of pattern overlap, degree of vertical symmetry, relative pattern surface area and contour density. The degree of pattern overlap was measured by calculating the percentage of pixels, which were identical at the same position in a pair of patterns. A value close to one indicates a high match of the compared patterns, whilst a value approaching zero indicates the patters were dissimilar. The similarity in degree of vertical symmetry was determined by drawing a vertical line in the center of the pattern and comparing each pixel on one side with the mirrored pixel on the opposite side to calculate the sum of deviation. A small value complies with a high degree of vertical symmetry. The relative pattern surface area was calculated as the ratio of the total number of black and white pixels. Finally, patterns were compared in terms of their contour density, measured as the ratio of pattern edge length and pattern area. For both relative pattern surface area and contour density, a high value indicates a high match of the compared patterns. All measures were done in Adobe Photoshop CS4 Extended.

### Scanning behavior

To analyze the pollinator’s behavior during pseudocopulation, 10 males of *Eucera berlandi* approaching an *O*. *heldreichii* flower were filmed in the field (near Neapolis, Crete, Greece, 35°15'N, 25°38'E) from above with a digital video camera (Sony DCR-SR50, Tokyo, Japan) at a rate of 25 frames s^-1^. The filmed area was 26 x 15cm and a 10 x 10mm grid on the ground was used as reference [25] for reconstructing the flight paths of individual males. The videos were analyzed frame by frame with the tracking software SkillSpector 1.3.0 (Video4coach, Svendborg, Denmark). From these data we measured the flight duration before landing, the length of stay on the labellum, and the time the male hovered in front of the flower before leaving. In addition, we calculated the scanning duration at different distances from the labellum and the angular deviation of the bees’ longitudinal body axis and the straight line between bee and flower.

### *Ophrys* pattern discrimination experiments with honeybees

We aimed to understand how the patterns of *O*. *heldreichii* are involved in the recognition and identification of individual flowers by the pollinator. One important prerequisite is that the bees are capable of perceiving the patterns, or, more specifically, the difference between patterns, despite the relatively low spatial resolving power of their compound eyes.

Since *E*. *berlandi* males are typically rare in the field and fly only for few weeks during mating season, we conducted the learning experiments with honeybees as an accessible and classic model of insect visual perception [40,41]. All bees possess apposition eyes and have comparable visual systems [42]. A honeybee workers eye consists of 5735 ± 143 ommatidia [43], while *E*. *berlandi* males possess 8354 ±237 per eye (mean ± SD; N = 5; Streinzer unpublished). The higher ommatidia number suggests a slightly higher visual acuity compared to honeybee workers (Land, 1997). Nevertheless, we anticipated useful information about the pollinator’s pattern discrimination abilities from our behavioral experiments. For a detailed discussion about the limitations of transferring conclusions from our experiments with honeybee workers to *E*. *berlandi* males see discussion below.

The standardized black-and-white patterns (see above) were chosen for the learning experiment. The patterns were printed on light grey paper cards and laminated in mat laminating pouches (54 x 86mm) to both enable washing (to remove potential scents), and to eliminate specular reflections. Four patterns of flowers from the same inflorescence (Fig 1B) and six patterns of flowers from different plants (Fig 1C) were randomly selected. For each of the treatment groups, one pattern was chosen as a target, whereas the remaining ones served as distractors. One of the distractor patterns was further randomly selected and kept for the final transfer test (see below). Two experimental groups were tested, since patterns of different plants and patterns of the same plant respectively were presented.

The experiments were carried out with 16 honeybees (8 bees per treatment group), which is consistent with bee psychophysics studies [44–49]. Bees were trained individually from a feeding dish with 0.1M sugar solution to visit a vertical rotating screen of 60cm diameter, where 1M sugar solution was offered for correct landings on freely rotating hangers with a landing platform [44,45,50]. This experimental set-up prevented position learning and allowed the bees to choose any visual angle in order to recognize the stimuli [51].

During pre-training only one rewarding target was presented on the screen for 8 visits, which is a form of absolute conditioning [52]. For each landing the bee was allowed to drink a 10μl drop of sugar solution on the landing platform and another drop on a plastic stick, presented right beside the platform. While the bee was moved away from the screen sitting on the stick, the position of the target was changed by rotating the screen. To prevent any impact of olfactory cues, the landing platforms were covered with a transparent film, which was renewed after each foraging bout when the bee returned to the hive.

Following pre-training, each bee was trained with differential conditioning [52] with two rewarding targets and two unrewarding distractors presented on the screen for the next 140 decisions. To increase motivation, a landing on the (wrong) distractor stimulus was punished with a drop of bitter tasting quinine solution that honeybees are unable to detect with olfactory cues [39,53,54]. After training, a non-rewarded learning test that consisted of 20 decisions was conducted to collect data that completely excluded any possible use of olfactory or spatial cues. After refresher training, which means rewarding the bees to maintain their motivation, the bees were finally tested with a non-rewarded transfer test with two targets versus two distractors of a novel pattern for another 20 decisions.

During its approach to a pattern, a bee’s response could be either correct (landing after approaching to the target stimulus or aborting an approach when approaching the distractor stimulus) or incorrect (landing on the distractor stimulus or aborting an approach to the target stimulus). A decision was considered as landing as soon as the bee was in contact with the stimuli or the landing platform. When the bee flew closer than 5cm to the stimuli and turned away afterwards without touching the stimuli or the landing platform, choices were counted as rejection[45].

To analyze such a complex decision making, signal detection theory has been proven useful in human psychology [55] but also to analyze behavior in bee learning experiments [45,56].

First, we calculated the probability of correct choices to the target stimulus (*Pc*, Eq 1) and incorrect choices to the distractor stimulus (*Pi*, Eq 2) for each training and test interval.

Pc=∑target landings/(∑target landings+∑target aborts) (Eq 1)

Pi=∑distractor landings/(∑distractor landings+∑distractor aborts) (Eq 2)

For each of these probabilities *Z* scores were calculated. The difference between the *Z* scores defines the variable *d’*, where a *d’* score of zero indicates chance performance and a value of 3.29 indicates perfect performance (for 10 decisions) [45]. To test, whether bee performance differed from chance, we calculated *d’* scores for each individual and then tested the observed distribution of *d’* scores against a value of zero using a one sample t-test. For convenience, we also present percent correct choices (target landings plus distractor aborts divided by total number of choices) as a more intuitive measure for performance. All statistical tests were performed on *d’* scores.

### Statistics

The statistical analyses were conducted using SPSS 15.0 (SPSS Inc.). To test if patterns of the same inflorescence are more similar than those of various plants a Mann-Whitney U-test was applied. For comparison between different populations a Kruskal-Wallis H-test was carried out. Differences between durations of approaching and scanning behavior were tested by a Mann Whitney U-test. To test whether honeybees are able to discriminate between flower patterns, we compared the *d’* scores for the last training block, the unrewarded test and the transfer test against an expected value of zero using a one-sample t-test (see above). All p-values above 0.05 were considered as statistically non-significant. When multiple comparisons were made, α-level was adjusted using Bonferroni correction.

## Results

### Pattern analysis for quantification of similarity

**Fig 2:**
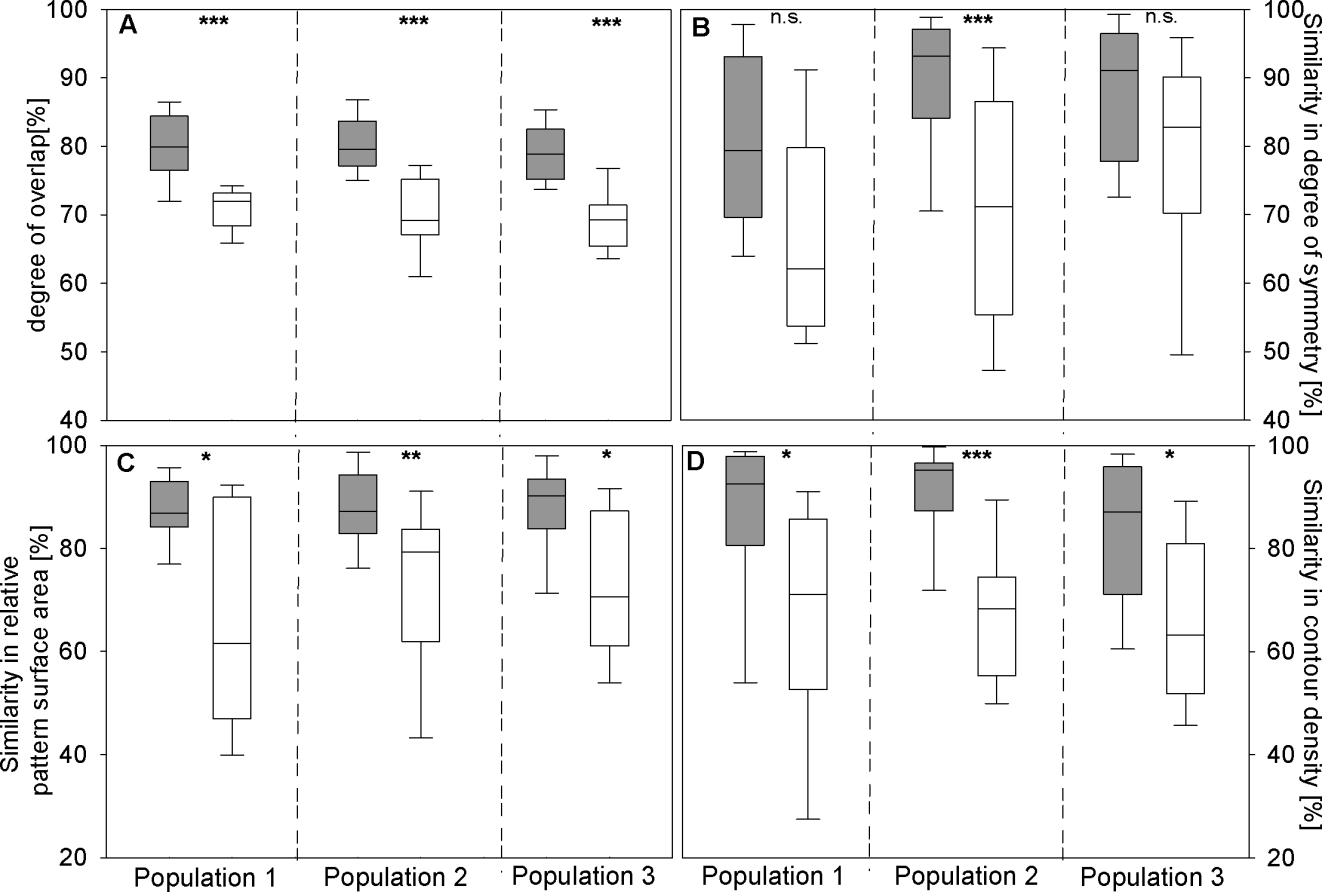
Degree of similarity among patterns from three *O*. *heldreichii* populations. Each box consists of 15 comparisons of randomly chosen flowers of the same (dark grey) or from different inflorescences (light grey). Four different features were compared: (A) pattern overlap, (B) pattern symmetry, (C) relative pattern surface area, and (D) contour density. Boxes represent inter-quartile range with median values. Stars indicate statistical differences after Bonferroni correction (***, p<0.00025; **, p<0.0025; *, p<0.0125; n.s., not significant).

Labellum patterns of flowers from the same inflorescence showed a significantly higher degree of similarity (pattern overlap, degree of symmetry, relative pattern surface area, contour density) than patterns from different plants *within* each of the three populations (Fig 2). Interestingly, however, pattern similarity did not differ statistically *between* the populations, both for pattern similarity within the same plant (pattern overlap (H(2) = 4.155, p = 0.125), degree of symmetry (H(2) = 5.277, p = 0.071), relative pattern surface area (H(2) = 0.313, p = 0.855), contour density (H(2) = 2.439, p = 0.295) and between different plants (pattern overlap (H(2) = 1.869, p = 0.393), degree of symmetry (H(2) = 4.155, p = 0.125), relative pattern surface area (H(2) = 1.018, p = 0.601), contour density (H(2) = 0.0664, p = 0.967)).

### Scanning behavior

**Fig 3:**
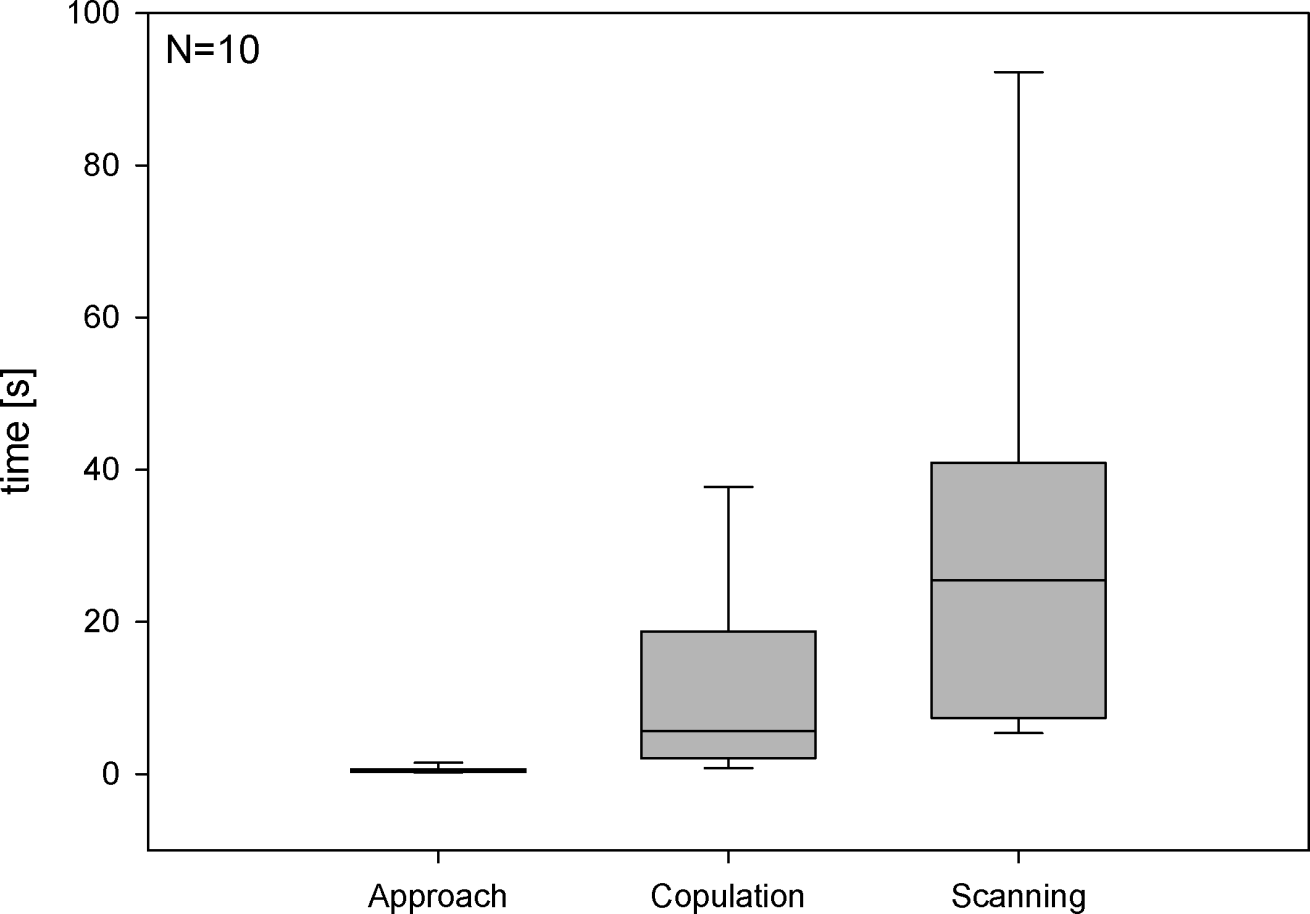
Duration of approach, copulation and post-copulation scanning behavior of *Eucera berlandi* males on an *Ophrys heldreichii* flower. Boxes represent inter-quartile range with median values, whiskers represent the 5 and 95 percentiles.

**Fig 4:**
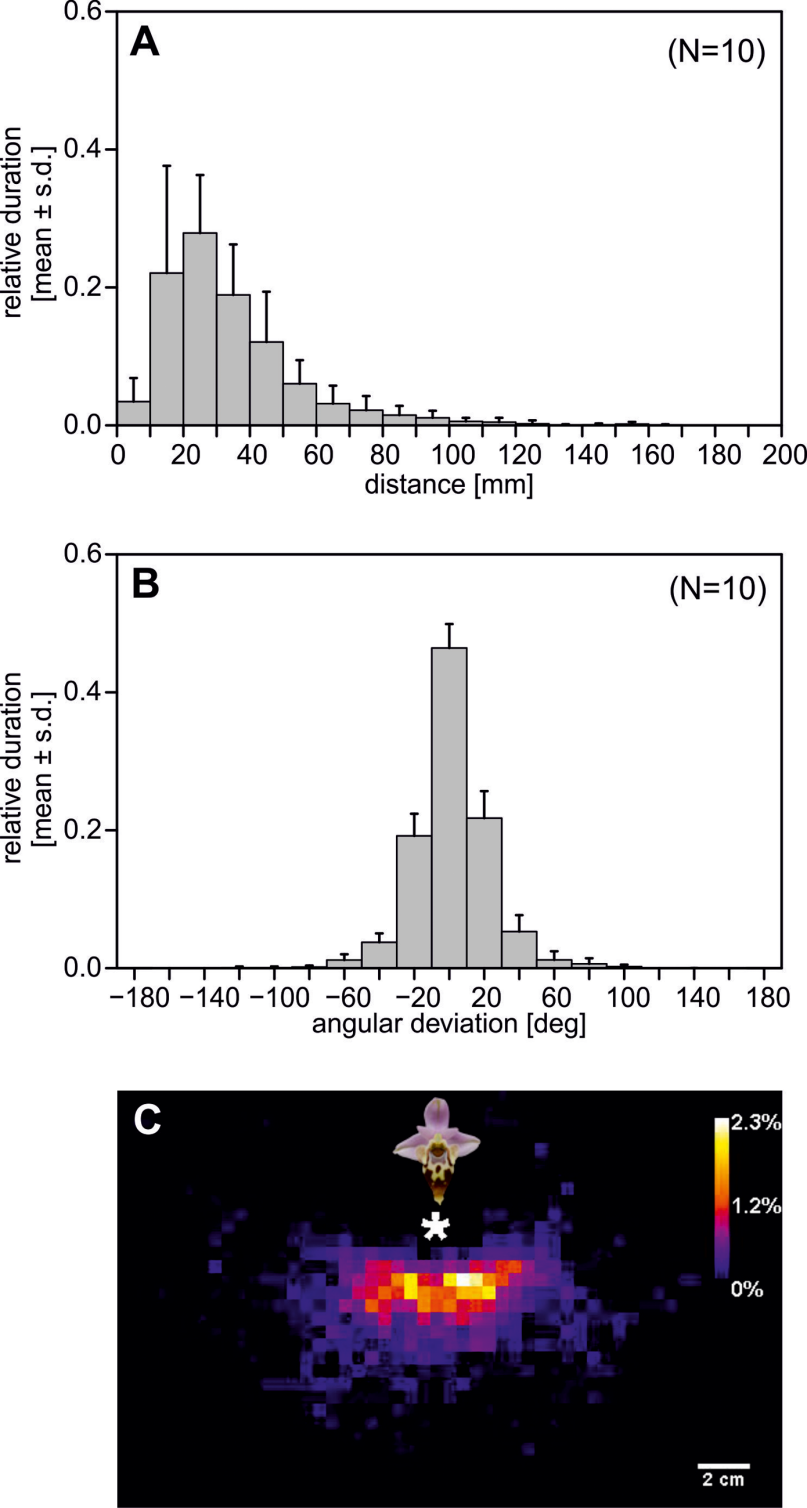
Scanning behavior of *Eucera berlandi* males after pseudocopulation on an *Ophrys heldreichii* flower (N = 10 flights). (A) Relative scanning duration at various distances from the flower. (B) Angular deviation of the bees’ longitudinal body axis and the straight line between bee and orchid flower. (C) Bee position during a scanning flight in one representative trial in top view (sequence length 100 seconds). Colors represent the probability of presence in each 5x5mm pixel. The asterisk marks the position of the flower. Error bars show the standard deviation from the mean in (A) and (B).

Males approached the flower at high velocity (< 1s flight time in the observed area of 390cm^2^) and attempted to copulate with the labellum for 5.7s (median; min. 0.7s, max. 38.9s; Fig 3). After breaking off the copulation, males did not immediately leave the flower but hovered in front of it, facing the labellum, for 25.4s (median; min. 5.7s, max. 97.2s; Figs 3 and 4C). The durations of approaching and post-copulation scanning behavior differed significantly (T = 55.0, p<0.001). During this “scanning behavior” they swung to the left and right while constantly facing the pattern head-on (Fig 4B) and kept an median flight distance of 2.0–4.1cm (range of medians; N = 10 Fig 4A) to the labellum, which corresponds to visual angles of 16°-30°as viewed by the males (Fig 4).

### *Ophrys* pattern discrimination experiments with honeybees

**Fig 5:**
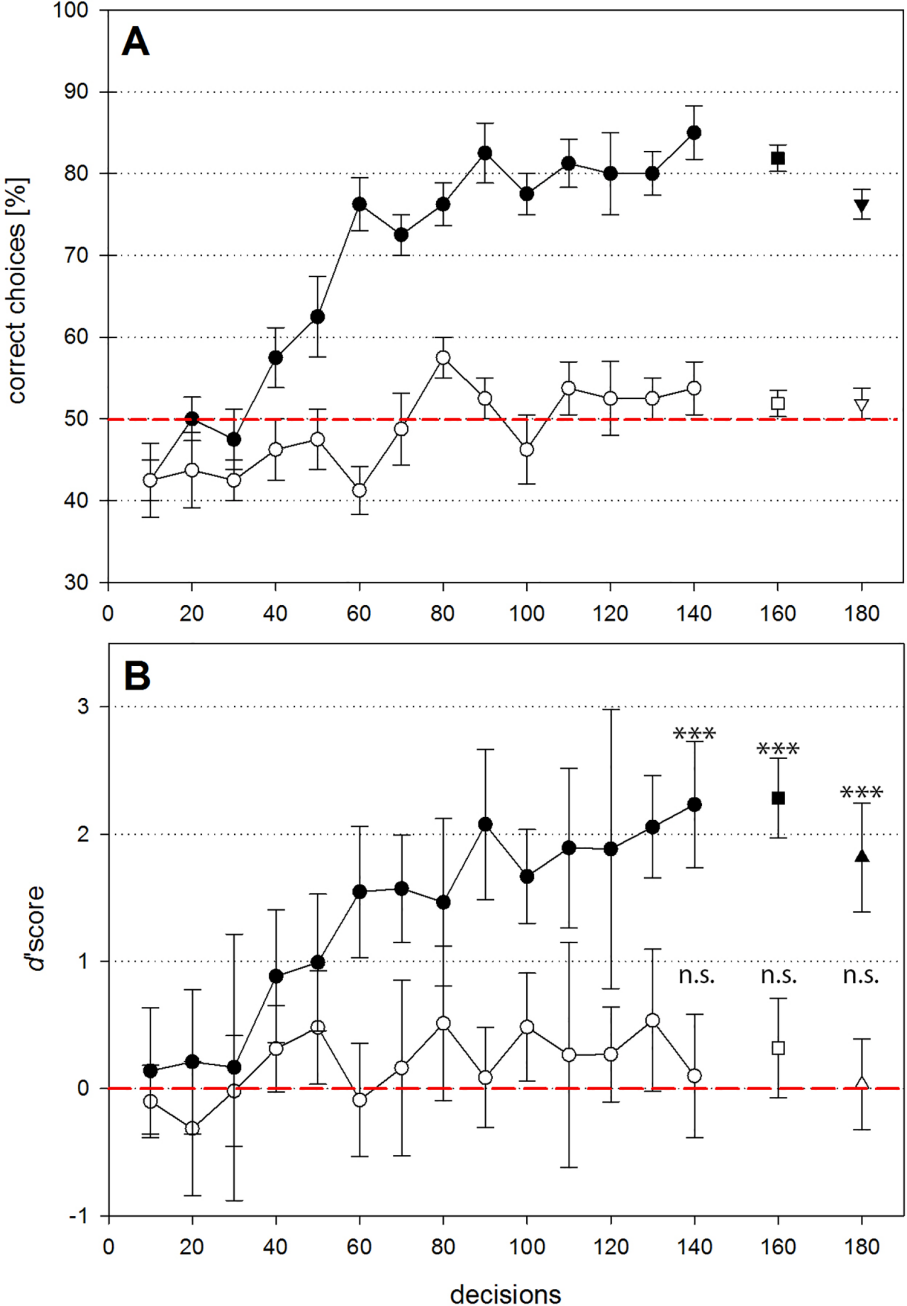
Honeybee performance during pattern discrimination experiments on a rotating screen. (A) Percent correct choices (target landings plus distractor aborts divided by total number of choices) during the pattern discrimination experiment. (B) Distribution of *d*’scores. Perfect discrimination would result in *d*’scores of 3.29, whereas values of zero indicate chance level performance. Filled circles show discrimination of pattern from different plants, open circles show discrimination of patterns from the same inflorescence. The square (after 160 decisions) symbolizes the performance in the unrewarded test, the triangle (after 180 decisions) the performance in the transfer test. Stars indicate statistical difference from chance (***p<0.001; n.s., not significant). Statistical tests were performed on *d’* scores only (see Results).

While patterns of flowers from different plants could be learnt and discriminated to a high level of accuracy consistently above 70% by one group of bees (*d’*: 2.23±0.50; mean±s.d.; one sample t-test, t = 12.74, d.f. = 7, P<0.001; Fig 5), the group of bees trained with patterns of flowers from the same plant, were not able to distinguish between patterns of the ‘same’ inflorescence, even after a long training period of 140 decisions (*d’*: 0.10±0.49; one sample t-test, t = 0.58, d.f. = 7, P = 0.58; Fig 5). The performance during the unrewarded test was significantly better than expected by random choice when patterns of flowers from different plants were presented (*d’*: 2.28±0.31; one sample t-test, t = 20.54, d.f. = 7, P<0.001), but at chance level when patterns of flowers from the same plant were presented (*d’*: 0.32±0.39; one sample t-test, t = 2.30, d.f. = 7, P = 0.055; Fig 5).

The additional transfer tests revealed that bees did not only learn to discriminate between the training stimuli, but were able to choose the correct pattern in a novel stimuli combination when the distractor came from a novel plant (*d’*: 1.82±0.43; one sample t-test, t = 12.05, d.f. = 7, P<0.001), but not when the distractor was a novel pattern from the same plant (*d’*: 0.04±0.36; one sample t-test, t = 0.28, d.f. = 7, P = 0.79; Fig 5).

In both experiments it appears that bees chose more frequently a distractor stimulus than a rewarded target during the first training block (Fig 5). However, this is most likely due to the way decisions were scored rather than a preference for the distractor. Specifically; once a bee had chosen a *distractor* stimulus for the first time, she sometimes elected to land again on the platform, because she spent some time recognizing and becoming adjusted to the novel situation in the training procedure where now four stimuli were presented simultaneously (two targets and two distractors) in contrast to only one rewarded target (and no distractor) during pre-training. However, when she instead had chosen a *target* for the first time, she was immediately rewarded and removed from the disk by the plastic stick, which was counted as only one (correct) decision.

## Discussion

In the present study we tested whether the variation in bright and complex patterns on the labellum of *O*. *heldreichii* flowers can act as a visual cue that could promote learning by a pollinator after pseudocopulation, and thus be potentially beneficial for both the pollinator and the plant.

The results are surprising in many aspects since classical models of insect visual perception only suggested that bees could process coarse elemental cues but were not capable of fine discriminations [57–59]. However, recent work from several groups has questioned such models of insect visual perception since bees can learn very complex visual stimuli like human faces [44], classic paintings [46] or pictures of landscapes [45,47], and other insects like the wasp *Polistes fuscatus* can reliably use vision to recognise the faces of conspecifics [60–62]. The reason for such contradictions has not been clear, and the new evidence we present shows a clear biological case where insects benefit from such impressive vision capabilities; which in some cases approaches the limit of what mammalian brains can achieve with visual processing [46,48,49]. In particular, we show that the signal provider, *O*. *heldreichii* may receive a benefit by promote allogamy, whilst the fine spatial vision of bees potentially provides individuals with a capacity to learn beneficial or non-beneficial stimuli through experience.

**Fig 6:**
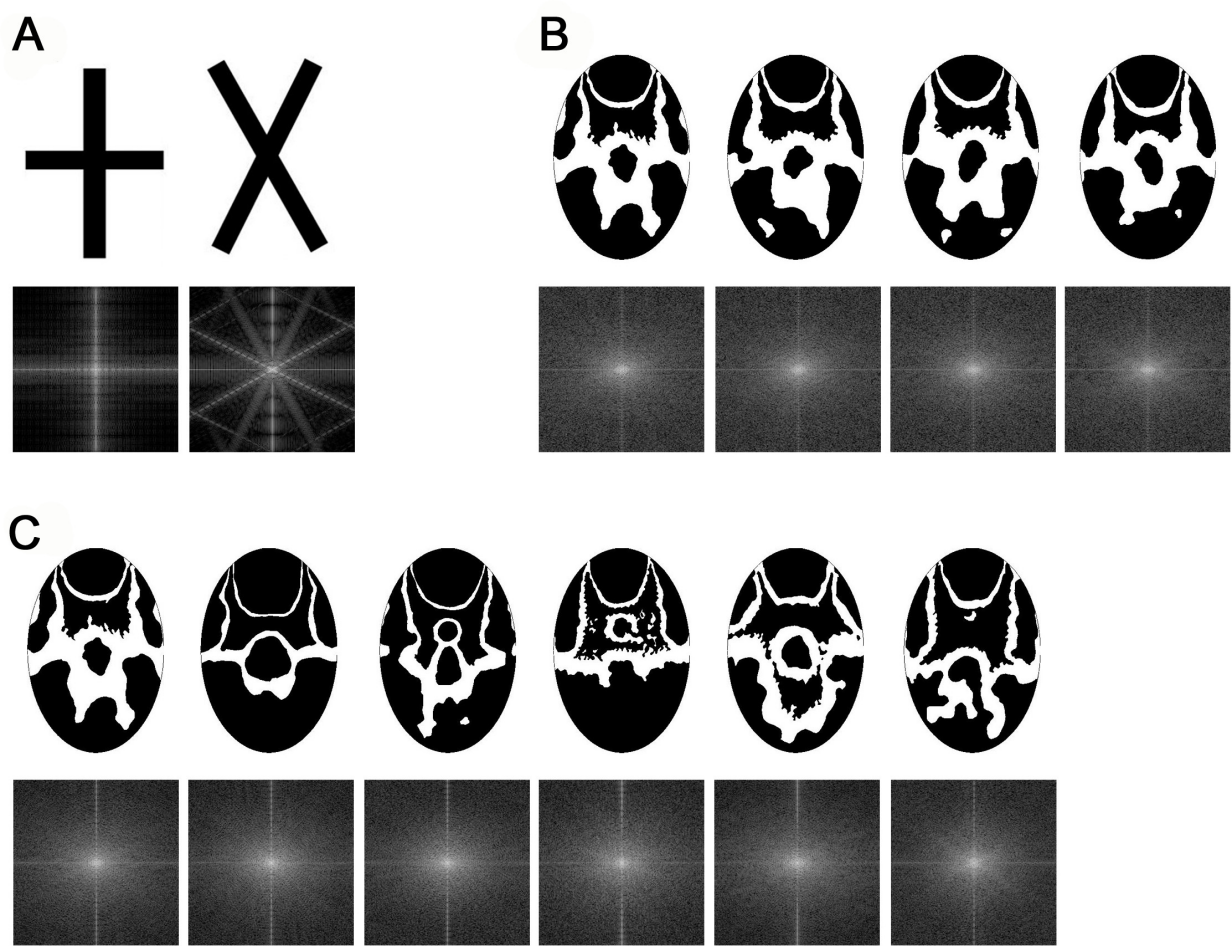
Fast Fourier Transform (FFT) of stimuli used in the honeybee experiments. Whereas the two types of crosses (A) differ widely in their FFT, the *Ophrys* pattern of same inflorescences (B) as well as those from different plants (C) show high similarity in their FFTs.

A variety of different theories have been developed to understand how insects like bees visually perceive the world [41,59,63,64]. Early theories were largely based upon the use of specific cues [59,65–67], but later work showed that bees can use both cues or even more sophisticated visual learning like configurations [48,68,69] and that these differences were due to both conditioning procedure and training length [52,70]. Indeed, recent work shows that attention is likely to be an important mechanism in bee choices since individual honeybees can learn complex hierarchical visual stimuli containing both local and global cues; and can modulate preferences towards either local or global cues depending upon experience or priming effects [71]. A comparison of labellum patterns in our experiments revealed a higher similarity within an inflorescence, whilst between inflorescences all tested cues revealed significantly higher variation (Fig 2). These potential floral cues are consistent with cues suspected to be involved in some forms of pattern recognition capability of bees [65,66,72]. Indeed the quantified higher similarity in these cues did result in more difficulties for honeybees learning the pattern discrimination tasks, suggesting our similarity specification is a valuable tool for evaluating how complex visual stimuli may be processed by bees. Another way that previous authors have quantified perceptual similarity of spatial stimuli is by measuring the Fast Fourier Transforms (FFT) of images [45–48]. We thus also measured the FFT of *Ophrys* patterns compared to rather simple 'cross' stimuli that differed greatly in spatial content of information. In comparison to the FFT for the cross stimuli, the various *Ophrys* labellum patterns show high level of similarity in their FFTs (Fig 6), thus also supporting our metric of similarity using a method comparable to previous work.

Field observations of *E*. *berlandi* males approaching an *O*. *heldreichii* flower demonstrated that the pollinators, attracted by the scent, very quickly approached the flower without taking time to inspect the labellum pattern, suggesting that the pattern is not involved in male attraction [26]. However, once an individual bee was deceived by the plant, it hovered in front of the flower and appeared to inspect the detail of the labellum pattern (Fig 4D). This observed scanning behaviour was previously reported in several studies about shape discrimination experiments with honeybees [73,74] and provides support for previous assumptions that, following pseudocopulation, males try to memorize the plant’s pattern and scent to prevent or reduce any further perceptual errors [11,13,14,20]. These previous findings are consistent with our current results of pattern discrimination experiments with honeybees using the rotating screen, which showed that bees were able to discriminate between patterns of different plant individuals, but failed to discriminate between patterns from flowers of the same inflorescence. The bees’ performances in the unrewarded tests are in agreement with the performance of the last training block, thus allowing us to dissect that it is possible to make the discrimination solely on the basis of visual cues since the unrewarded tests were with fresh stimuli that exclude all olfactory cues. However, we have to act with caution when generalizing the results obtained with our honeybee model to other bee species, but we believe that our major conclusions hold also for *E*. *berlandi* males. Due to a slightly higher ommatidial number *E*. *berlandi* males should possess a slightly higher spatial resolving power [36] and thus should be able to discriminate fine patterns at least as well as honeybees do. Learning in honeybees was slow and it took at least 50 decisions to significantly discriminate the two patterns (Fig 5). In contrast, *E*. *berlandi* males usually visit orchids only a few times before they lose interest [75] and thus need to learn much faster. In addition, *E*. *berlandi* males are faced with aversive learning, whereas in honeybees we used appetitive conditioning. The learning speed and strength of an association is modulated by a number of factors. For example, the costs of a honeybee worker for choosing the wrong stimulus is low (few seconds of foraging time) since she can easily fly to the next stimulus. In contrast, competition among *E*. *berlandi* males for virgin females is extremely high due to high male numbers and few females which mate only once. Thus the decision to land on an orchid flower may prevent a male from detecting one of the rare females and might cause significant fitness consequences for the male. Our observations, that *E*. *berlandi* males spend a long time scanning, but honeybees made pretty fast decisions indicates different attentional levels and also supports this hypothesis. Due to these potentially high fitness costs learning speed is most likely higher in mate-seeking males than in bees foraging for food.

The learning and avoidance of individual flowers by the pollinator would lead to negative frequency-dependent selection, which may explain the high variation of labellum patterns among *O*. *heldreichii* flowers, since patterns, which are more similar to each other, are probably more often rejected in comparison to different ones [4,76]. As a consequence, flowers with rare patterns would receive more visits which probably leads to higher reproductive success. Previous work has documented a comparable variation in another modality, in particular odour signals of *Ophrys sphegodes*. Minimal differences in the odour components led to avoidance of previously visited flowers, but not of others [20]. The pollinator, *Andrena nigroaenea* males seem to reliably learn and memorize an individual flower odour bouquet and avoid future visits after an unsuccessful copulation event.

Recently, Sedeek et al. [77], investigated trait variation and reproductive isolation in four closely related species of *Ophrys*. They report a high intra-specific variation in labellum speculum shape and suggest, in line with our working hypothesis, that this variation may function in learning and subsequent avoidance of already visited flowers. However, no detailed information is available with respect to the difference between inter-plant and between-plant speculum shape variation.

The higher similarity of labellum patterns within rather than among plants is consistent with selection, but also consistent with neutrality. It is likely that flowers of the same plant are more similar due to genetic and developmental constraints, as it had been shown for flower colour and pattern [78]. It would be important to examine in future studies if the pattern similarity with a plant in *O*. *heldreichii* is due to developmental constraints or indeed caused by selection by the pollinator [79,80]. In the latter scenario we predict that rare pattern types receive more pollinator visits and thus have a higher reproductive success than more common pattern types.

Aversive learning of individual flower traits may be an important strategy by sexually deceptive orchids in general, to reduce geitonogamy and thus promote allogamy. Recent investigations in the Australian sexually deceptive orchid genus *Chiloglottis* suggest that high levels of outcrossing may help to circumvent the costs, i.e. reduced seed germination and growth, of geitonogamous pollination [81]. Most species in the genus *Ophrys* possess inconspicuous labella without highly contrasting patterns. Therefore we hypothesize that the presence of these strong visual signals might be correlated to the importance of the visual system in the pollinator’s mating behaviour, as was already assumed for the coloration of the sepals in some *Ophrys* species, including *O*. *heldreichii* [25]. *Eucera* males are highly visually guided during mate search and thus visual signature learning may be more important in this system compared with the olfactory signature learning that was shown for the less visually guided males of *Andrena nigroaenea* [20]. However, pattern variation is also found in *Ophrys* species with a less conspicuous labellar speculum [75]. This suggests that visual pattern learning may also contribute to individual signature learning in less visually guided bee species. It will be necessary to investigate further *Ophrys* species in future studies to examine if the presence of labellum pattern variation is correlated to pollinators with distinct visual systems.

## Supporting Information

S1 Fig Experimental setup for learning experiments with honeybees. Two target and two distractor stimuli were presented on hangers on a vertically rotation screen of 60cm diameter. The shown enlarged stimuli were used in a pre-experiment.
(TIF)

S1 Table Raw data. (XLSX)
