# Supplementary figures and images for: Functional Significance of Labellum Pattern Variation in a Sexually Deceptive Orchid (Ophrys heldreichii): Evidence of Individual Signature Learning Effects
Source: PLoS One. 2015 Nov 16;10(11):e0142971. doi: 10.1371/journal.pone.0142971 (PMC4646623; doi:10.1371/journal.pone.0142971)

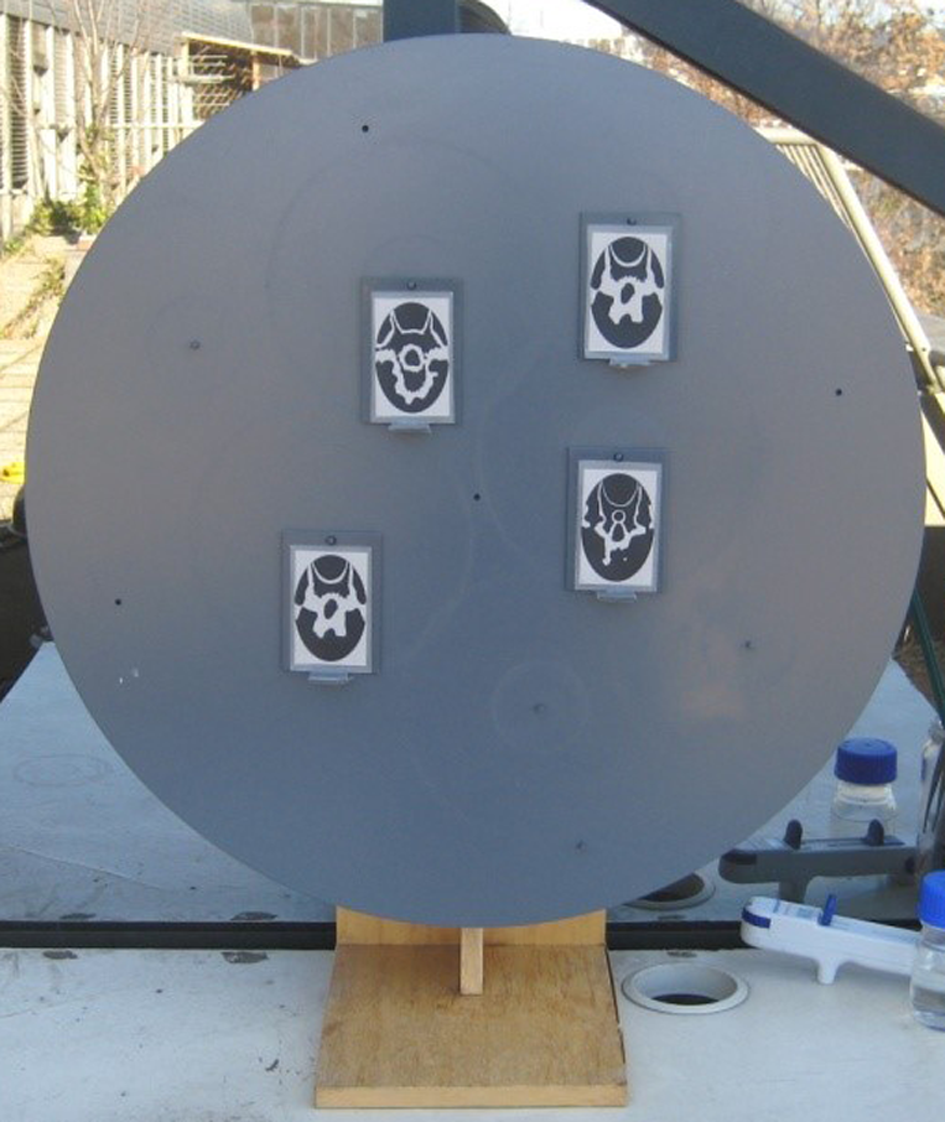

Supplement: S1 Fig — Two target and two distractor stimuli were presented on hangers on a vertically rotation screen of 60cm diameter. The shown enlarged stimuli were used in a pre-experiment. (TIF) [file pone.0142971.s001.tif]
